# Submaximal exercise training improves mitochondrial efficiency in the gluteus medius but not in the triceps brachii of young equine athletes

**DOI:** 10.1038/s41598-017-14691-4

**Published:** 2017-10-30

**Authors:** Sarah. H. White, Lori K. Warren, Chengcheng Li, Stephanie E. Wohlgemuth

**Affiliations:** 1 Department of Animal Sciences, College of Agricultural and Life Sciences, University of Florida, Gainesville, USA; 2 Present Address: Department of Animal Science, College of Agriculture and Life Sciences, Texas A&M University, College Station, USA

## Abstract

We tested the hypothesis that, similar to humans and rodents, exercise training would enhance mitochondrial (Mt) biogenesis and function in skeletal muscle of young horses. Twenty-four Quarter Horse yearlings were randomly assigned to either submaximal exercise training or no forced exercise (untrained). Biopsies were collected from the gluteus medius and triceps brachii before and after 9 wk of treatment. Citrate synthase activity was lower (P < 0.0001) and cytochrome *c* oxidase activity per Mt unit was higher (P < 0.0001) in gluteus compared to triceps, but neither changed over the trial period. From wk 0 to 9, intrinsic Mt respiration (P_**CI**_, P_**CI+II**_; P = 0.008) and electron transport capacity (E_**CI**+**II**_; P = 0.01) increased, and LEAK-related flux control factor (FCF_L_; P = 0.02) decreased in both muscles. After 9 wk of training, gluteus muscle exhibited higher (P < 0.05) intrinsic P_**CI**_, P_**CI**+**II**_, E_**CI**+**II**_, and FCF_CI_ and FCF_**CI+II**_, and lower FCF_L_ (P = 0.0002). Mitochondrial content did not change from wk 0 to 9, and also not in response to submaximal exercise training. Improvements in Mt function were most directly related to ongoing growth of horses independent of muscle group, and training further enhanced Mt function in the gluteus medius.

## Introduction

Exercise training has been shown to enhance mitochondrial (Mt) biogenesis and improve Mt function in human and rodent models^1^. These adaptations have not been clearly elucidated in horses, especially at a young age when it is common for horses to enter performance training. Considered the powerhouse of the cell, mitochondria are vital to maintain energy production during exercise. Thusly, focus should be placed on Mt function, more specifically the Mt electron transport system (ETS), when evaluating adaptations to exercise training^2^. In humans and rodents, the individual complexes of the Mt ETS respond differently to exercise training, collectively improving the efficiency of energy production and decreasing the production of potentially harmful reactive oxygen species^3,4^. By comparison, investigation of Mt function in horses is in its infancy, and no published research evaluating Mt function in young horses undergoing exercise training exists.

The objectives of this study were to determine if a low- to moderate-intensity training protocol, typical of what would be used in the industry to prepare young pleasure performance horses for competition, would affect Mt biogenesis and function in two separate muscles of differing fiber type composition. We hypothesized that training would enhance Mt biogenesis as well as improve Mt function, and that these adaptations would be augmented in the gluteus medius compared to the triceps brachii.

## Results

### Mt enzyme activities

**Figure 1:**
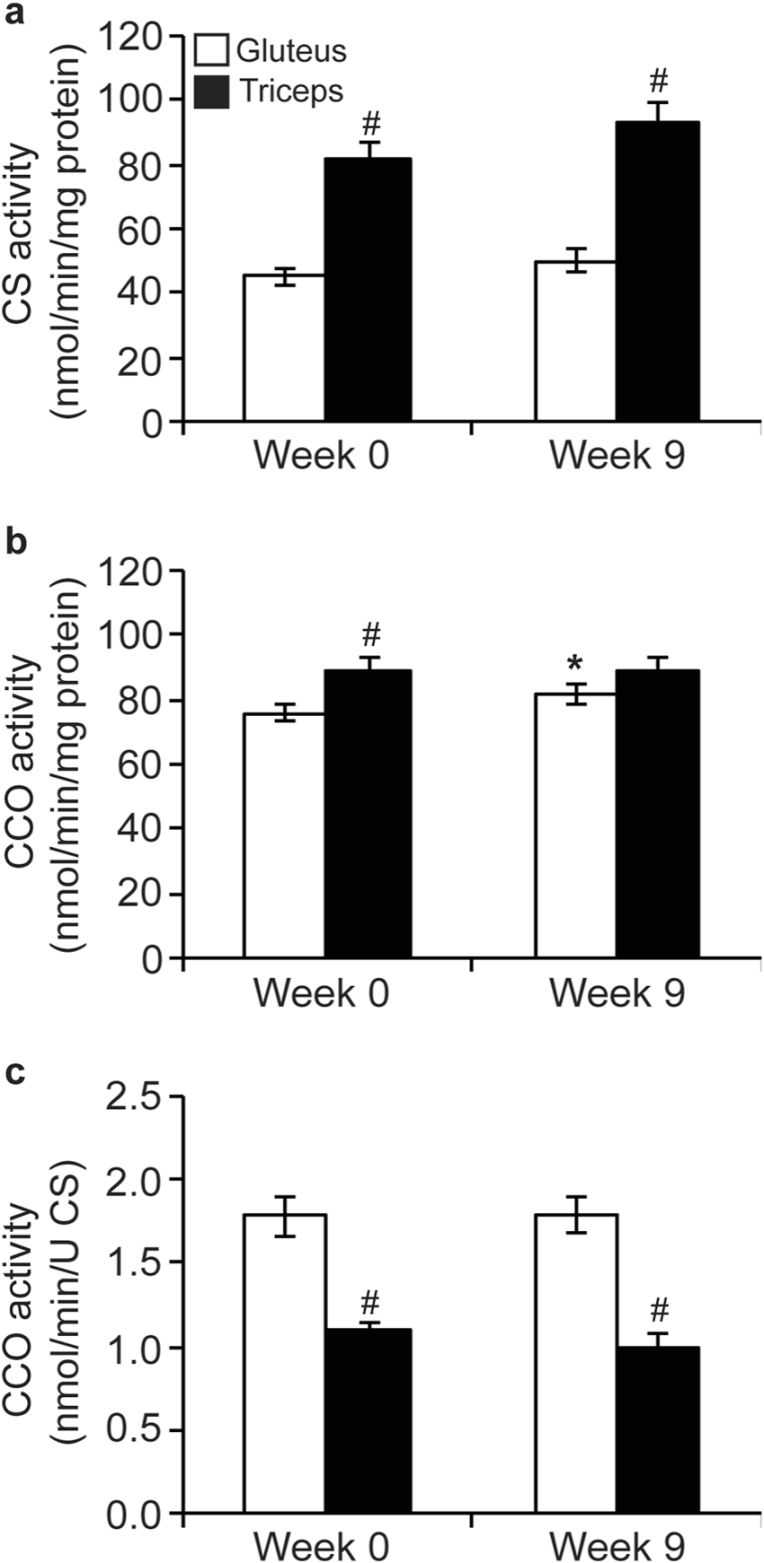
Activities of citrate synthase (CS) (**a**); and cytochrome *c* oxidase (CCO) normalized to total protein (**b**) and normalized to CS activity (**c**) in the gluteus medius (GM; n = 24) and triceps brachii (TB; n = 12) of horses before and after a 9 wk observation period (mean ± SEM). Data were analyzed using a mixed model ANOVA with repeated measures. Due to a lack of effect of training, data from untrained and trained horses were combined. Overall effects of time (P = 0.2, P = 0.1, P = 0.9), muscle (P < 0.0001, P = 0.001, P < 0.0001), and time × muscle (P = 0.6, P = 0.1, P = 0.9), for activities of CS (**a**), CCO relative to total protein (**b**), and CCO relative to CS activity (**c**), respectively. *Within muscle group, week 0 different than week 9 (P < 0.05). ^#^Within time point, GM different than TB (P < 0.05).

Training had no effect on citrate synthase (CS) or cytochrome *c* oxidase (CCO) activity in either muscle (data not shown). Therefore, data from untrained and trained horses were pooled for the subsequent enzyme activity analyses (Fig. 1). Citrate synthase activity in the triceps was 2-fold greater (P < 0.0001) compared to the gluteus at both wk 0 and 9, and did not change over time in either muscle (Fig. 1a). When expressed relative to total protein, CCO activity was higher in the triceps than the gluteus at wk 0 (P = 0.001) and increased only in the gluteus from wk 0 to 9 (P = 0.02), resulting in similar CCO activity between muscles at wk 9 (Fig. 1b). When expressed relative to CS activity, CCO activity was lower in the triceps than the gluteus at both wk 0 and 9 (P < 0.0001), and did not change over time in either muscle (Fig. 1c).

### Mt respiration in two different muscles from growing horses

**Figure 2:**
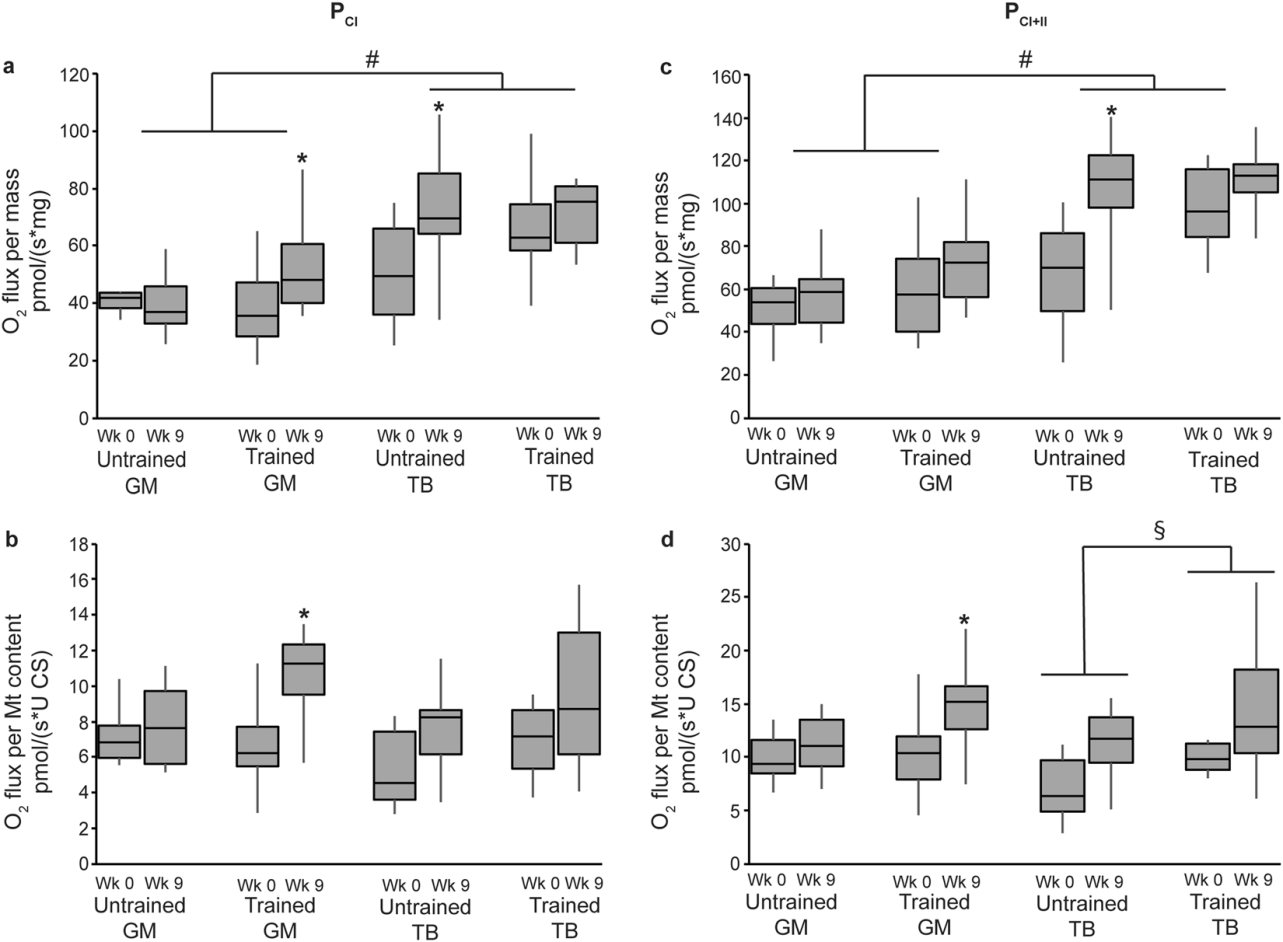
Integrative (**a**,**c**) and intrinsic (**b,d**) mitochondrial ADP-stimulated respiration (P) in the gluteus medius (GM) and triceps brachii (TB) of horses before and after 9 wk of either receiving no forced exercise (Untrained; n = 6 GM, n = 6 TB) or being exercise trained (Trained; n = 17 GM, n = 6 TB). (**a**) P with complex I substrates (P_CI_), glutamate and malate, relative to tissue weight; (**b**) P_CI_ relative to mitochondrial content; (**c**) P_CI_ plus succinate, a complex II substrate (P_CI+II_), relative to tissue weight; and (**d**) P_CI+II_ relative to mitochondrial content. Data were analyzed using a mixed model ANOVA with repeated measures. Boxplots represent the median (line in center of box) and the 1^st^ and 3^rd^ quartiles, and error bars represent the 95% confidence interval. Overall effects for panels a, b, c, and d, respectively, of time (P = 0.02, P = 0.004, P = 0.002, P = 0.002), muscle (P < 0.0001, P = 0.3, P < 0.0001, P = 0.5), training (P = 0.2, P = 0.02, P = 0.03, P = 0.005), time × muscle (P = 0.5, P = 0.9, P = 0.2, P = 0.4), time × training (P = 0.9, P = 0.2, P = 0.4, P = 0.4), muscle × training (P = 0.8, P = 0.7, P = 0.5, P = 0.5), and time × muscle × training (P = 0.05, P = 0.3, P = 0.2, P = 0.6). *Within training and muscle group, wk 0 different than wk 9 (P < 0.05). ^#^Across untrained and trained, GM different than TB (P < 0.05). ^§^Across GM and TB, untrained different than trained (P < 0.05).

Respiration data was analyzed both relative to tissue wet weight (integrative Mt respiration) and normalized to Mt content (intrinsic Mt respiration). Maximum ADP-simulated respiration (OXPHOS) with complex I substrates, glutamate and malate (P_CI_), and with the addition of succinate, a complex II substrate (P_CI+II_), was higher in the triceps than the gluteus at the tissue level (integrative, P < 0.0001 for both; Fig. 2a and c), but not at the Mt level (intrinsic; Fig. 2b and d).

**Figure 3:**
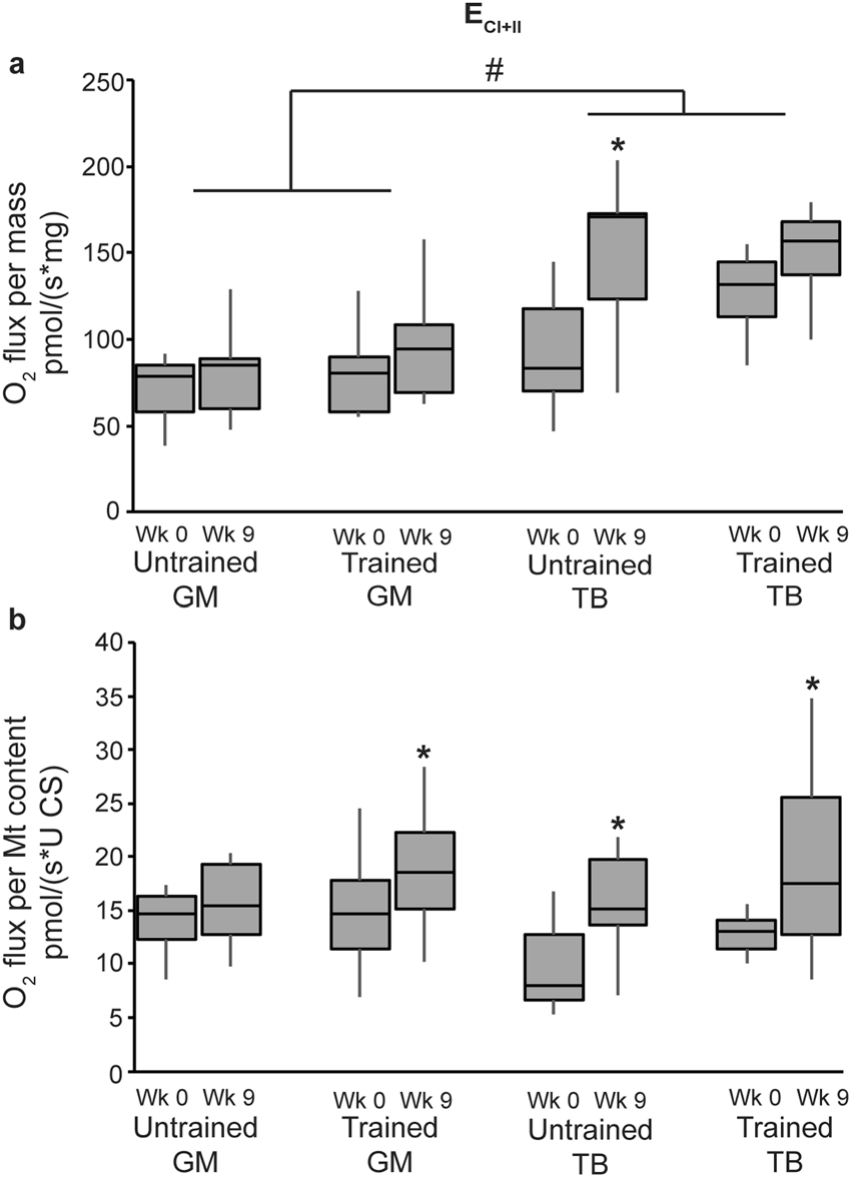
Electron transport system capacity (E_CI+II_) relative to tissue weight (**a**) and relative to mitochondrial content (**b**) in the gluteus medius (GM) and triceps brachii (TB) of horses before and after 9 wk of either receiving no forced exercise (Untrained; n = 6 GM, n = 6 TB) or being exercise trained (Trained; n = 17 GM, n = 6 TB). Data were analyzed using a mixed model ANOVA with repeated measures. Boxplots represent the median (line in center of box) and the 1^st^ and 3^rd^ quartiles, and error bars represent the 95% confidence interval. _CI+II_ = glutamate + malate + succinate. Overall effects for panels (a) and (b), respectively, of time (P = 0.002, P = 0.004), muscle (P < 0.0001, P = 0.1), training (P = 0.1, P = 0.03), time × muscle (P = 0.07, P = 0.2), time × training (P = 0.3, P = 0.9), muscle × training (P = 0.7, P = 0.4), and time × muscle × training (P = 0.2, P = 0.4). *Within group, wk 0 different than wk 9 (P < 0.05). ^#^Across untrained and trained, GM different than TB (P < 0.05).

Integrative maximum electron transport capacity (E_CI+II_) was higher in the triceps than the gluteus (P < 0.0001; Fig. 3a), whereas intrinsic E_CI+II_ was similar between the gluteus and triceps (Fig. 3b).

### Mt respiration and exercise

Analysis of integrative Mt respiration revealed that the submaximal exercise training protocol resulted in higher P_CI+II_ (P = 0.03) independent of muscle group, but did not affect any other integrative respiration variables. Relative to Mt content, P_CI_ (P = 0.02), P_CI+II_ (P = 0.005), and E_CI+II_ (P = 0.03) were higher in trained compared to untrained horses (Figs 2 and 3) independent of muscle group. Further, within the triceps, intrinsic P_CI+II_ was higher in trained than untrained horses (P = 0.03; Fig. 2d).

Overall, integrative Mt OXPHOS (P_CI_ and P_CI+II_; P = 0.02 and P = 0.002, respectively) and E_CI+II_ (P = 0.002) increased from wk 0 to 9 independent of training regimen; the same response was noted with intrinsic Mt function (P = 0.004, P = 0.002, and P = 0.004 for P_CI_, P_CI+II_, and E_C__I__+__II_, respectively; Figs 2 and 3).

Relative to tissue weight (integrative Mt respiration), the gluteus from trained horses showed lower LEAK respiration (3.18 ± 0.51 versus 0.686 ± 0.51 pmol/(s*mg) for wk 0 and 9, respectively; P = 0.002) and higher P_CI_ (P = 0.005; Fig. 2a) at wk 9 compared to wk 0. During this time, P_CI_ (P = 0.02; Fig. 2a), P_CI+II_ (P = 0.002; Fig. 2c) and E_CI+II_ (P = 0.001; Fig. 3a) had increased in the triceps of untrained horses, but no changes occurred in the triceps of trained horses. Intrinsic Mt respiration in the horses’ muscles demonstrated more consistent patterns over time. The gluteus of trained horses exhibited increased P_CI_ (P = 0.0003; Fig. 2b), P_CI+II_ (P = 0.005; Fig. 2d), and E_CI+II_ (P = 0.05; Fig. 3b), and decreased LEAK (0.57 ± 0.09 versus 0.13 ± 0.09 pmol/(s*U CS) for wk 0 and 9, respectively; P = 0.001) at wk 9 compared to wk 0. Additionally, intrinsic E_CI+II_ increased from wk 0 to 9 in the triceps of untrained (P = 0.05) and trained horses (P = 0.03). However, no further differences were detected between wk 0 and 9 in the gluteus of untrained horses, nor the triceps of untrained or trained horses.

**Figure 4:**
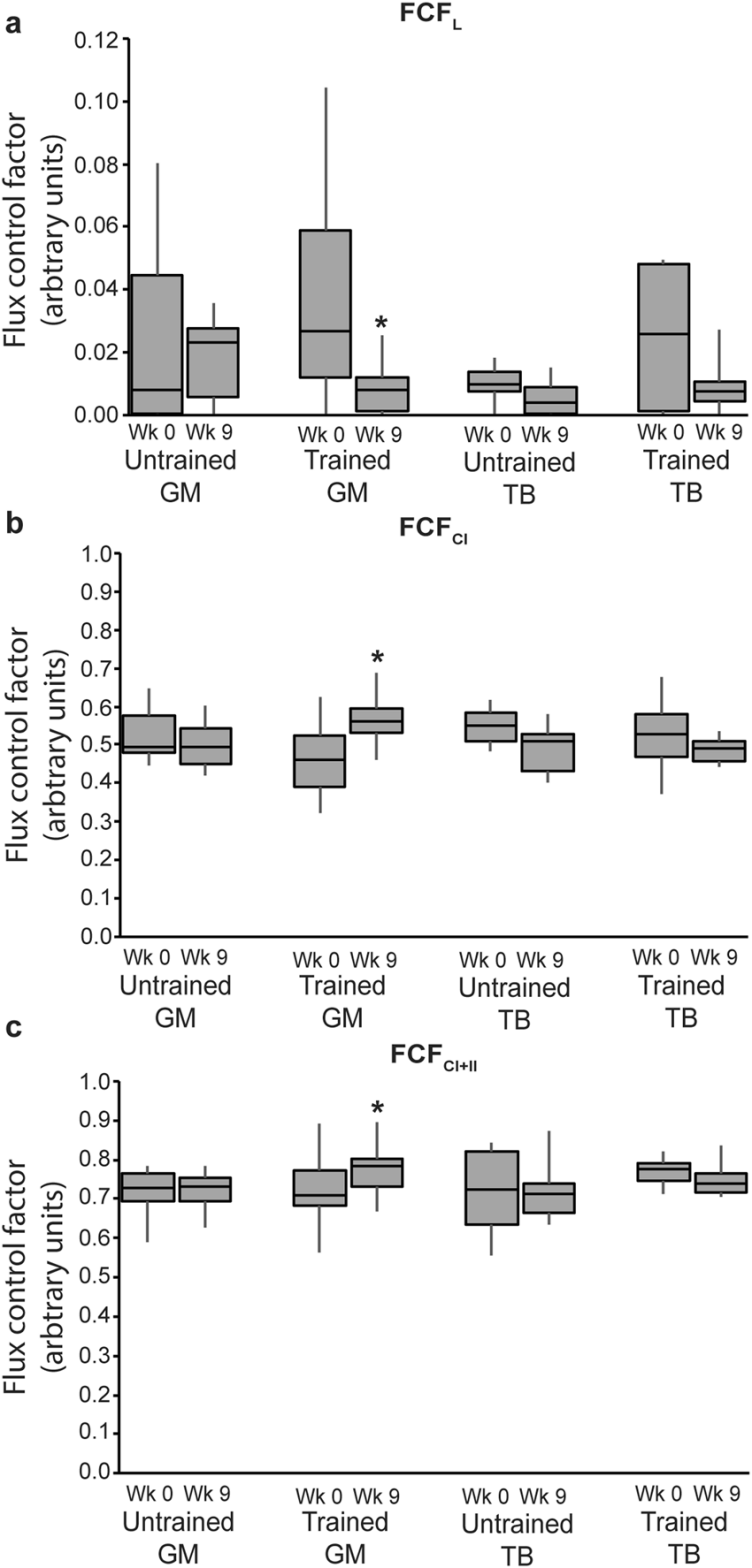
Flux control factor (FCF) in the gluteus medius (GM) and triceps brachii (TB) of horses before and after 9 wk of either receiving no forced exercise (Untrained; n = 6 GM, n = 6 TB) or being exercise trained (Trained; n = 17 GM, n = 6 TB). (**a**) _L_ = Leak (glutamate + malate, no ADP); (**b**) _CI_ = glutamate + malate + ADP; and C) _CI+II_ = glutamate + malate + ADP + succinate. Data were analyzed using a mixed model ANOVA with repeated measures. Boxplots represent the median (line in center of box) and the 1^st^ and 3^rd^ quartiles, and error bars represent the 95% confidence interval. Overall effects for panels (a), (b), and (c), respectively, of time (P = 0.01, P = 0.8, P = 0.9), muscle (P = 0.06, P = 0.9, P = 0.9), training (P = 0.5, P = 0.8, P = 0.04), time × muscle (P = 0.6, P = 0.03, P = 0.2), time × training (P = 0.1, P = 0.06, P = 0.5), muscle × training (P = 0.6, P = 0.7, P = 0.6), and time × muscle × training (P = 0.7, P = 0.2, P = 0.6). *Within group, wk 0 different than wk 9 (P < 0.05).

The flux control factors (FCF) for LEAK, P_CI_, and P_CI+II_ (which represent the fraction of respiration at that particular respiratory state of the maximum electron transport system capacity, E_CI+II_) are presented in Fig. 4. Overall, FCF_L_ (P = 0.01) decreased from wk 0 to 9, but there was no difference between muscle groups or training status (Fig. 4a). While FCF_CI_ was not different due to muscle group or training (Fig. 4b), FCF_CI+II_ was higher in trained compared to untrained horses (P = 0.04; Fig. 4c). Investigation of individual muscle groups revealed that, similar to oxidative capacities, FCF_L_ decreased (P = 0.0002; Fig. 4a), and FCF_CI_ (P = 0.0001; Fig. 4b) and FCF_CI+II_ (P = 0.04; Fig. 4c) increased in the gluteus of trained horses but all FCFs were unchanged from wk 0 to 9 in the gluteus of untrained horses and in the triceps of untrained and trained horses.

## Discussion

This study is the first to investigate Mt adaptations to submaximal exercise training in skeletal muscles of young American Quarter Horses. The triceps of 2-year-old horses had greater CS activity than the gluteal muscle in the current study. Citrate synthase activity correlates with Mt density^5^, which is typically greatest in type I (slow twitch, oxidative) muscle fibers, followed by type IIa (fast twitch, oxidative), and lowest in type IIx (fast twitch, glycolytic)^6,7^. Based on higher CS activity, it can be surmised that the triceps of 2-year-old Quarter Horses in the current study had greater Mt density than the gluteus medius. Direct support comes from our previous analysis of myosin heavy chain (MyHC) isoform distribution in triceps and gluteus muscles from this same group of horses. At sampling depths of 5 cm, the triceps contained around 10% MyHC-I, 33% MyHC-IIa, and 55% MyHC-IIx, compared to the gluteus with 3% MyHC-I, 21% MyHC-IIa, and 75% MyHC-IIx^8^. A similar relation of type I fiber distribution between triceps and gluteus was reported for Dutch Saddle Horses at a sampling depth of 5 cm (~25% versus ~10% for the triceps and gluteus, respectively)^9^. In contrast, others have found fiber type distribution to be similar between these two muscles in horses. For example, Andrews and Spurgeon^10^ reported percentages of type I, IIa, and IIx to be 22, 36, and 42%, respectively, for the triceps, and 23, 36, and 40%, respectively, for the middle gluteus across several breeds, including Thoroughbreds, Quarter Horses, Arabians, Paso Finos, and Shetland Ponies. However, the authors reported the sampling depth as “superficial”, which might explain the discrepancy with our results^11^.

Both chronic and acute exercise have been shown to stimulate Mt biogenesis in skeletal muscle^12–16^. In our study, the unchanged CS activity suggests that Mt content in either muscle was not affected by submaximal exercise training. In support, a previous study conducted by our group revealed that 14 wk of a similar exercise training protocol was insufficient to elicit a change in CS activity in the middle gluteus of 2-year-old Quarter Horses^17^. It is possible that the exercise regimen employed in our study induced both Mt biogenesis and turnover^18^, thereby masking the exercise effect on Mt content. Alternatively, the absence of an apparent exercise effect could be due to the nature of the training protocol, either being too short in duration or of too low intensity. In contrast to the current study, mature Andalusian horses undergoing low intensity (25–30% V_LA_4) exercise training 5 d/wk for 60 min/d had a 20% increase in CS activity in the gluteus medius, as well as a 16% increase in the area occupied by type IIa fibers and a 17% increase in the ratio of type IIa: IIx after 12 wk of training^19^. Similarly, Arabian horses undergoing endurance training at a higher intensity (~80% VO_2_ max) for 50–80 min/d, 4 d/wk had an increased relative area occupied by type I and IIa fibers after 12 wk of training^20^. A greater exercise intensity or a longer duration may be necessary to increase Mt density in this population, as Quarter Horses tend to have a greater percentage of type IIa and IIx fibers than either Andalusian or Arabian horses^21^.

Activities of both CS and CCO, as well as cytochrome *c* protein levels have been used as biomarkers for Mt content^22^. Larsen *et al*.^5^ more recently determined that CCO activity does not correlate as well with Mt content (as determined by electron microscopy) as CS activity, but is more strongly associated with OXPHOS capacity. When the two muscles were compared, we found that integrative CS activity (relative to tissue protein) was about 2-fold higher in the triceps than in the gluteus, while integrative CCO activity was similar between muscles. Interestingly, intrinsic CCO activity (normalized to CS activity as biomarker of Mt content) was significantly lower in the triceps compared to the gluteus. This suggests that a higher Mt content in the triceps compensated for a lower intrinsic Mt function.

This study was the first to use HRR to examine specific aspects of OXPHOS and electron transport in the triceps and gluteus of young Quarter Horses undergoing submaximal exercise training. Similar to CCO activity, respiration data varied based on whether integrative or intrinsic Mt function was considered. Integrative OXPHOS and ETS capacities were greater in the triceps than the gluteus, but intrinsic Mt respiration measurements were similar between muscle groups. Both respiration and CCO activity suggest a similar or even lower intrinsic Mt function in the triceps compared to the gluteus, and the higher integrative Mt function observed in the triceps is likely a result of its greater Mt content.

Interestingly, muscle differences were less pronounced for integrative CCO activity than for E_CI+II_ respiratory capacity. While CCO is only one of several complexes of the electron transport system, E_CI+II_ capacity is a measure of integral Mt respiratory function, taking into account that the degree of respiratory capacity depends on the functionality of multiple protein complexes. Additionally, the greater integrative E_CI+II_ capacity of the triceps supports the hypothesis that compared to the gluteus, the triceps may have a greater ability to respond with higher electron flux and increased Mt energy production when necessary. The gluteus, on the other hand, may have a lower overall aerobic capacity. Research in other mammals indicated muscle-specific mitochondrial capacities were based on the muscle’s primary means of energy production^23,24^. Oxidative muscles have been shown to have greater mass-specific (integrative) oxidative capacities than glycolytic muscles, but lower or similar mitochondrial-specific (intrinsic) oxidative capacities compared to glycolytic muscles^25^, which is consistent with our results.

The submaximal exercise regimen employed in this study is representative of regimens used to train pleasure horses to be ridden, and not so much an exercise regimen in preparation for competitive performance. Our objective was to evaluate whether this common type of training affected muscle Mt function in young horses. Previous research in our lab has suggested improved fitness after 8 weeks of a similar submaximal exercise training protocol in 2-year-old Quarter Horses, as evidenced by lower resting heart rates and lower heart rates 30 min after a 2-h submaximal exercise test compared to untrained control horses^26^. In the current study, there was an overall effect of 9 wk of submaximal exercise training in improving *integrative* P_CI+II_, as well as *intrinsic* P_CI_, P_CI+II_ and maximum E_CI+II_ capacity independent of muscle type. However, training-specific *intrinsic* Mt adaptations were significant only in the gluteus, which showed elevated P_CI_, P_CI+II_ and E_CI+II_. Votion, *et al*.^27^ reported an increase in integrative P_CI_ in both the gluteus and triceps of mature Arabian horses following 10 wk of endurance training, but no difference in P_CI+II_. Further, E increased after training in the triceps but not in the gluteal muscle^27^. A subsequent study by Votion, *et al*.^28^ explored the differences in integrative Mt function in the triceps brachii of untrained, trained, and “competitive” adult horses. Untrained and trained horses had similar P_CI_, which was lower than in competitive horses; yet, both trained and competitive horses had higher P_CI+II_ and E compared to untrained horses^28^. The differences between the reports by Votion, *et al*.^27,28^ and our results suggest that mature, adult horses respond differently to exercise training than younger horses that are still growing. Further, there may be breed differences regarding adaptations to exercise, given that Arabians tend to have a more oxidative muscle profile than Quarter Horses^7,21^.

Exercise training regimens reported to affect Mt function differ between species, possibly due to differing lifespan and metabolic rates, which makes extrapolation from one species to another challenging. For example, in rats, as few as 10 d of treadmill running for 30 min/d was sufficient to increase both P_CI_ and P_CI+II_ in the gastrocnemius muscle^3^, and 10 wk of endurance cycling training in men resulted in elevated P_CI+II_ and E_CI+II_ in the vastus lateralis muscle^4^. The similar response to endurance training in men and to low-intensity exercise training in horses reported here suggest that the horse may be a better model for future human exercise research than the rat.

Lastly, one of the most intriguing findings from this study was the response to exercise training in the gluteus. We found significant increases in OXPHOS (P_CI_ and P_CI+II_) and E_CI+II_, and in fractional control (FCF) of CI and CI + II in the gluteus medius following 9 wk of exercise training, suggesting that the exercise regimen employed in this study improved maximum Mt energy production and Mt efficiency in this muscle group. We expected to see adaptations in the triceps because of its greater percentage of type I fibers that can be stimulated by aerobic exercise. With aerobic exercise training, muscle composition gradually shifts to a more oxidative phenotype (type I and IIa)^2^. However, the triceps exerts a more postural role than the gluteus, and it is possible that Mt capacity of the triceps provided sufficient energy for this exercise intensity, or that this muscle group was not sufficiently stimulated by this type of training to undergo fiber type switch or Mt adaptations. The main function of the gluteus medius, on the other hand, is to produce impulsion for movement of the hind leg^29^, which performs most of the propulsion during forward movement^30^. Therefore, the gluteus is more likely to respond to an increase in physical activity that places emphasis on a forward movement.

In conclusion, we found that the triceps brachii had greater Mt density than the middle gluteal muscle in 2-year-old American Quarter Horses, but intrinsic Mt function was similar between the two muscle groups prior to exercise training. The submaximal exercise training protocol was not sufficient to induce Mt biogenesis, but our data indicate that Mt function was improved by exercise training, specifically in the gluteus medius. Overall, growth itself had the greatest impact on Mt function in this group of horses, indicating progressive improvements in Mt capacity for aerobic energy production as the horse grows into maturity. This novel method of quantifying Mt capacity in horse skeletal muscle might not only inform horse owners and trainers about training regimens for young horses, but it may become clinically important as it is to date in human medicine^31^ to help identify myopathies or other muscle bioenergetic pathologies for veterinarians and horse owners alike.

## Materials and Methods

### Horses

Twenty-four American Quarter Horse yearlings (14 fillies and 10 geldings) with a starting mean ± SEM age of 21 ± 0.2 mo and bodyweight (BW) of 410 ± 9 kg were utilized in this study. Horses undergoing exercise training (n = 18; 11 fillies and 7 geldings) were housed in two separate 3-ha pastures in groups of 9 horses each at the Institute of Food and Agricultural Sciences (IFAS) Horse Teaching Unit in Gainesville, FL. These horses were stabled individually in 3.7 × 3.7 m stalls 3 d/wk for approximately 8 h/d. Non-exercised horses (n = 6; 3 fillies and 3 geldings) were group-housed in a 16-ha pasture at the IFAS Equine Sciences Center in Ocala, FL. This study was reviewed and approved by the IFAS Animal Research Committee at the University of Florida, and all methods were carried out in accordance with the approved Animal Use Protocol.

For at least 4 wk before the start of the study, all horses were maintained on the same diet formulated to meet or slightly exceed nutrient requirements for growing horses in light work^32^. The daily ration of individual horses consisted of 1% BW (DM) of a commercial concentrate and *ad libitum* access to Coastal bermudagrass hay. Although horses were housed on pasture, pasture forage was dormant during the study period and thus nutrient intake from grazing was deemed negligible. This same feeding program was continued during the 9-wk study period. Adjustments were made to the amount of concentrate fed based on changes in BW, which were assessed weekly using a livestock scale accurate to ±0.5 kg (MP800, Tru-Test, Inc., Mineral Wells, TX). Nutrient analysis was performed on all feedstuffs prior to the start of the study by Equi-Analytical Laboratories (Ithaca, NY) using standard analytical methods. The nutrient composition of the diet is presented in Table 1.

**Table 1:** Nutrient composition the diet.

Nutrient^a,b^	Bermudagrass Hay	Grain Concentrate
DE (Mcal/kg)	0.89	1.59
Crude fat (%)	1.7	8.7
Crude protein (%)	10.1	15.8
NDF (%)	67.7	23.2
ADF (%)	37.4	9.7
Ca (%)	0.65	0.88
P (%)	0.25	0.70
Zn (mg/kg)	37	123
Cu (mg/kg)	6	43

^a^Values presented on a 100% dry matter basis. ^b^DE = digestible energy; NDF = neutral detergent fiber; ADF = acid detergent fiber.

### Exercise training and sample collection

For at least 5 wk prior to the start of the study, all horses were group-housed on 16-ha pastures and received no forced exercise. Following the first sample collection (wk 0), horses in the trained group underwent submaximal exercise training 3 d/wk for 30–45 min/d consisting of walk, trot, and canter in a round pen or while being ridden.

Biopsies were taken as described previously (8) alternately from the right or left gluteus medius (gluteal) muscle of all horses (n = 24) and from the triceps brachii (triceps) of a subset of the trained horses (n = 6; 3 fillies and 3 geldings) and all untrained horses (n = 6) before (wk 0) and after 9 wk of exercise training. Biopsy site was prepared and biopsies were collected as previously published^8^. At each sampling time, approximately 75 mg of wet muscle tissue was placed in 1 mL ice-cold relaxing solution (BIOPS^27^; 10 mM Ca-EGTA buffer, 0.1 μM free calcium, 20 mM imidazole, 20 mM taurine, 50 mM K-MES, 0.5 mM dithiothreitol, 6.56 mM MgCl_2_, 5.77 mM ATP, and 15 mM phosphocreatine; pH 7.1) and stored at 4 °C until high-resolution respirometry (HRR) was performed (within 24 h post-collection). At every sampling interval, an additional 300 mg tissue was flash frozen in liquid nitrogen and stored at −80 °C until further analysis.

### Mitochondrial Enzyme Activities

For measurement of enzyme activities, frozen skeletal muscle samples were pulverized in liquid nitrogen (BioPulverizer, BioSpec Products, Inc., Bartlesville, OK), homogenized in sucrose homogenization buffer (20 mM Tris, 40 mM KCl, 2 mM EGTA, and 250 mM sucrose, pH 7.4) with 0.05% detergent (n-Dodecyl β-D-maltoside; Sigma D4641), sonicated (F60 Sonic Dismembrator, Fisher Scientific, Waltham, MA), and centrifuged at 11,000 *g* for 3 min. The supernatant was stored at −80 °C until further analysis of citrate synthase (CS) and cytochrome *c* oxidase (CCO) activities.

Enzymatic activities were measured as previously described^17,33^ using a microplate reader (Synergy HT, BioTek Instruments, 237 Winooski, VT, USA). An 80-fold dilution of muscle homogenate was analyzed for CS and CCO activities. Briefly, CS activity was assessed at 412 nm by measuring the linear rate of reaction of free CoA-SH with DTNB; CCO activity was determined by measuring the linear rate of oxidation of fully reduced cytochrome *c* at 550 nm.

Enzyme activities in muscle homogenates were normalized to protein content, determined using the Bradford Protein Assay Kit (Thermo Scientific, Rockford, IL). Using CS activity as a proxy for total mitochondrial content in the sample^34^, CCO activity was further normalized to mitochondrial content.

### High-Resolution Respirometry

#### Permeabilized muscle fiber preparation

Freshly collected muscle biopsy samples were placed in ice-cold BIOPS and fat and connective tissue removed. Muscle fibers were then prepared and permeabilized as previously described^8,35^. Permeabilized fibers were immediately used for HRR.

### High-resolution respirometry of permeabilized muscle fibers

Permeabilized fibers (2–3.5 mg *W*_w_) were added to each respirometer chamber of the Oxygraph-2k (O2k; Oroboros, Innsbruck, Austria) containing 2 mL of MiR06 (MiR05 + 5 μL 280 U/mL catalase) and 20 mM creatine^8^ and maintained at 37 °C. Oxygen concentration (μM) and oxygen flux per muscle mass (pmol O_2_ ● s^−1^ ● mg *W*_w_^−1^) were recorded with the DatLab software (Oroboros, Innsbruck, Austria). Throughout the entire substrate-uncoupler-inhibitor titration (SUIT) protocol, hyperoxic O_2_ concentrations (200–500 μM O_2_) were maintained by titration of H_2_O_2_ (100 mM) to prevent O_2_ limitation^28^.

**Figure 5:**
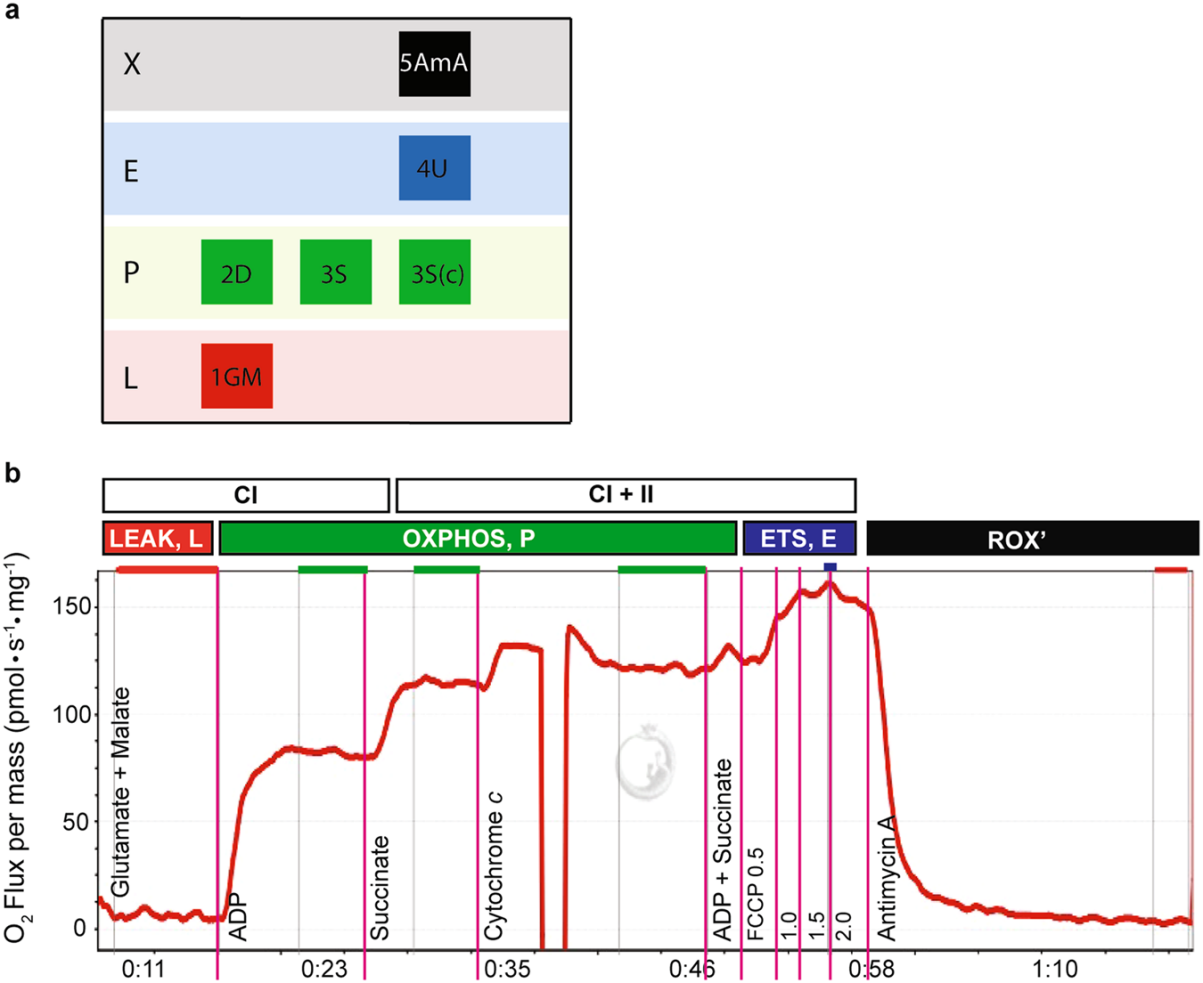
Experiment design (**a**) and example output (**b**; DatLab software by Oroboros, Innsbruck, Austria) from a high-resolution respirometry experiment on the Oxygraph-2k. The y-axis shows O_2_ flux per mass (pmol ▪ s^−1^ ▪ mg^−1^), with time on the x-axis. The SUIT protocol for this study was as follows: glutamate + malate (10 mM and 2 mM; LEAK, state 2), ADP (2.5 mM; P_CI_), succinate (10 mM; P_CI+II_), cytochrome *c* (10 μM), ADP + succinate (2.5 mM and 10 mM), 0.5 μM titrations of FCCP (E_CI+II_), and antimycin A (2.5 μM; ROX). O_2_ concentration was maintained between 200 and 500 pmol/mL throughout the experiment.

Oxygen flux and respiratory states were determined with the following SUIT protocol described previously for equine skeletal muscle^8^ (Fig. 5): 1) glutamate (10 mM) and malate (2 mM) to support electron flow through complex I (CI) of the electron transport system (LEAK respiration, L); 2) adenosine diphosphate (ADP; 2.5 mM) to stimulate respiration (OXPHOS, P_CI_); 3) succinate (10 mM) to support convergent electron flow through complex II (CII) of the electron transport system (P_CI+II_); 4) additional ADP (2.5 mM) and succinate (10 mM) to evaluate whether OXPHOS capacity could be increased any further, indicating that OXPHOS was measured at saturating ADP concentrations; 5) cytochrome *c* (cyt *c*; 10 μM) to assess outer mitochondrial membrane integrity (samples with responses to cyt *c* greater than 15% were excluded from the dataset); 6) uncoupler p-trifluoromethoxyphenyl carbonyl cyanide (FCCP; 0.5 μM steps) to assess maximum electron transport system capacity (E_CI+II_); 7) Antimycin A (2.5 μM), an inhibitor of complex III, to measure residual oxygen flux (ROX) independent of the ETS. Throughout the SUIT protocol, a stable O_2_ flux was recorded for approximately 5 min prior to the next titration. Flux control factors were calculated by dividing O_2_ consumption rate with each substrate by maximal O_2_ consumption (E_CI+II_). This yielded the following formulas: FCF_L_ = LEAK$\div$ E_CI+II_, FCF_CI_ = P_CI_ $\div$ E_CI+II_, and FCF_CI+II_ = P_CI+II_ $\div$ E_CI+II_. Respiration measurements were evaluated both relative to tissue wet weight and per Mt unit (normalized to CS activity in the sample)^34^.

### Statistical Analyses

All data were tested for normality and subsequently log-transformed before analysis when not normally distributed. Differences in CS and CCO activities and mitochondrial respiration measurements were analyzed by mixed model analysis of variance (Proc Mixed function, SAS version 9.3, SAS Institute Inc., Cary, NC) with repeated measures, where time served as a repeated variable. Compound symmetry or heterogeneous compound symmetry were selected as the covariate structure based on Akaike Information Criteria (AIC) and finite-sample corrected Akaike Information Criteria (AICC) for best model fit. Time, training, muscle, and all interactions were included in the model as fixed effects, and horse within treatment served as random effect. Data were expressed as the mean ±SE, with the exception of mitochondrial respiration data presented in figures, which were presented as boxplots that included the median, upper and lower quartiles, and 95% confidence intervals. Outliers were statistically identified and excluded from the data set, which resulted in n = 17 horses in the trained GM group for mitochondrial respiration analyses. Differences were considered significant at P ≤ 0.05.

### Data Availability

The datasets generated during and/or analysed during the current study are available from the corresponding author on reasonable request.
